# Enhanced Flame Retardancy in Ethylene–Vinyl Acetate Copolymer/Magnesium Hydroxide/Polycarbosilane Blends

**DOI:** 10.3390/polym14010036

**Published:** 2021-12-23

**Authors:** Tiefeng Zhang, Chunfeng Wang, Yongliang Wang, Lijun Qian, Zhidong Han

**Affiliations:** 1 School of Materials Science and Chemical Engineering, Harbin University of Science and Technology, Harbin 150040, China; zhangtiefeng79@163.com (T.Z.); yongliangwang@hrbust.edu.cn (Y.W.); zhidong.han@hrbust.edu.cn (Z.H.); 2 Engineering Laboratory of Non-halogen Flame Retardants for Polymers, Beijing Technology and Business University, Beijing 100048, China; 3 Key Laboratory of Engineering Dielectrics and Its Application, Ministry of Education, Harbin University of Science and Technology, Harbin 150080, China

**Keywords:** ethylene–vinyl acetate copolymer, magnesium hydroxide, polycarbosilane, flame retardant, thermal degradation

## Abstract

A polymer ceramic precursor material—polycarbosilane (PCS)—was used as a synergistic additive with magnesium hydroxide (MH) in flame-retardant ethylene–vinyl acetate copolymer (EVA) composites via the melt-blending method. The flame-retardant properties of EVA/MH/PCS were evaluated by the limiting oxygen index (LOI) and a cone calorimeter (CONE). The results revealed a dramatic synergistic effect between PCS and MH, showing a 114% increase in the LOI value and a 46% decrease in the peak heat release rate (pHRR) with the addition of 2 wt.% PCS to the EVA/MH composite. Further study of the residual char by scanning electron microscopy (SEM) proved that a cohesive and compact char formed due to the ceramization of PCS and close packing of spherical magnesium oxide particles. Thermogravimetric analysis coupled with Fourier-transform infrared spectrometry (TG–FTIR) and pyrolysis–gas chromatography coupled with mass spectrometry (Py–GC/MS) were applied to investigate the flame-retardant mechanism of EVA/MH/PCS. The synergistic effect between PCS and MH exerted an impact on the thermal degradation products of EVA/MH/PCS, and acetic products were inhibited in the gas phase.

## 1. Introduction

Ethylene–vinyl acetate copolymer (EVA) brings much convenience to daily life and industrial manufacturing due to its excellent dielectric properties and weather resistance [1,2]. As an important matrix resin, EVA is widely used in the wire and cable industry. The development of halogen-free flame-retardant EVA compounds for wires and cables has been an important topic from the perspective of science and technology. Some halogen-free flame retardants are essential in these formulations, such as hydroxycarbonates [3], magnesium hydroxide (MH) [4], aluminum trihydrate (ATH) [5], and other metallic hydroxides [6,7]. Considering that the flame-retardant effect mainly comes from their endothermic decomposition and the barrier role of the decomposed products [8], the flame-retardant properties of the compounds show strong dependence on the loading levels of these flame retardants. Generally, high loading levels (more than 50 wt.%) are required in order to meet the flame retardant demands, which deteriorate the overall performance of the flame-retardant compounds. The surface modification of the flame retardants [9,10] becomes a means to reduce the influence of high loading and improve the mechanical properties of the compounds [11]. Fiber [12] and compatibilizers [13] were also investigated to improve the mechanical properties and balance the flame retardancy of the compounds.

Many efforts have been made to seek more effective ways to increase the flame retardant efficiency of MH or ATH. Some functional additives were studied to reveal synergistic effects with MH and ATH in flame-retardant polyolefin compounds [14,15,16,17,18,19,20,21,22,23,24], such as silica [14], graphene [15,16,17], carbon nanotubes [18], nanoclay [19], hollow glass beads [20], melamine and melamine salts [21], zinc borate [22], phosphorus [23], and organosilicon [24]. These additives showed positive effects on the combustion behaviors of the composites [15,16,17,18,19,20,21,22,23,24], while the thermal degradation behaviors of polyolefin seemed not to be influenced [20,21,22]. The surface structure design of MH was also reported to improve the flame retardancy and mechanical properties of flame-retardant EVA composites [25]. As widely accepted, the combustion behaviors are highly related to the char formed during combustion [18,22,23]. Meanwhile, the limiting oxygen index (LOI) at a moderate content of the synergists can be related to the char [26,27].

Polycarbosilane (PCS) was widely reported as a polymer ceramic precursor with a polymeric backbone composed of silicon atoms and difunctional organic groups that connect the silicon atoms [28]. According to our previous investigation [26,27], the positive effects of PCS were reported to enhance the flame retardancy of polyethylene (PE) with MH as an inorganic flame retardant. The ceramic char was revealed to function as a barrier to heat and mass transfer during the combustion. Considering the significant differences in the thermal degradation process between EVA and PE, attempts were made in this work to employ PCS as a synergistic agent to improve the flame retardancy of EVA/MH composites. The influence of PCS on the thermal degradation and combustion behaviors of EVA/MH were investigated. The flame-retardant mechanism was proposed on the basis of the char structure in the condensed phase and the volatiles in the gas phase. The results of this work could be used for reference in the research of halogen-free flame-retardant EVA, as well as polyolefins, when combined with our related works.

## 2. Materials and Methods

### 2.1. Materials

Ethylene–vinyl acetate copolymer (EVA) containing 26 wt.% vinyl acetate, with the trade name 7470 M (melting index of 4.0 g/10 min at 190 °C and 2.160 kg, density of 0.948 g/cm^3^, Vicat softening point of 48 °C) was supplied by Formosa Plastics Co., LTD., Ningbo, China. Magnesium hydroxide (MH, 5-C) with a particle size of 0.9 μm (D_50_) was obtained from Dandong Songyuan Chemicals Co., LTD., Dandong, China. Polycarbosilane (PCS) with a structure of [SiH(CH_3_)CH_2_]_n_ was used. PCS with a number-average molecular weight of ~1320 and a purity of 99% was purchased from Suzhou Sailifei Ceramic Fiber Co., LTD., Suzhou, China. The above materials were commercial products, and were used without further treatment.

### 2.2. Preparation of Composites

PCS-modified MH was prepared as described in [26]. The flame-retardant composites were prepared via the melt-blending method with a HAPRO rheometer (Harbin Hapro Electric Technology Co., LTD., Harbin, China.). The rheometer was preheated to 120 °C and kept isothermal for 10 min. EVA pellets were added to the mixing chamber and blended for 2 min at a rate of 40 rpm. Then, the flame retardants (MH or MH/PCS) were added and blended for 10 min to obtain the flame-retardant composites. The composites were hot-pressed to the sheets at 150 °C under 10 MPa for 5 min. The composites were named according to their composition, such as EVA/MH/PCS 50/49/1. The former part of the name (EVA/MH/PCS) represents the component abbreviation, while the latter (50/49/1) represents the content of the components by weight percentage.

### 2.3. Characterization

The limiting oxygen index (LOI) was measured on an oxygen index tester (HC-2, Nanjing Jiangning Analytical Instrument Factory, Nanjing, China) according to ASTM D2863, with the sample dimensions of 100 mm long, 6 mm wide, and 3 mm thick.

Thermogravimetric analysis (STA 449 F3, NETZSCH, Weimar, Germany) coupled with Fourier-transform infrared spectrometry (Nicolet 6700, Thermo Fisher Scientific, Waltham, MA, USA) was performed at a heating rate of 10 °C/min in a nitrogen flow of 3 L per hour; the volatiles were detected at a scan number of 30 and a resolution of 2 cm^−1^, from 4000 cm^−1^ to 400 cm^−1^.

The volatiles of pyrolysis were identified by pyrolysis–gas chromatography coupled with mass spectrometry (Py–GC/MS, QP-2010Ultra, Shimadzu, Kyoto, Japan).

The combustion behaviors were evaluated according to ISO 5660 on a CONE calorimeter (6810, Suzhou Yangyi Vouch Testing Technology Co., Ltd., Suzhou, China) at heat flux of 35 kW/m^2^, with square specimens of 100 mm long, 100 mm wide, and 3 mm thick. Several combustion parameters—namely, time to ignition (TTI), time to peak heat release rate (tpHRR), peak heat release rate (pHRR), total heat release (THR), and total smoke release (TSR)—were given. Furthermore, fire performance index (FPI), fire growth index (FGI), total heat release index (THRI), and total smoke production index (TSPI) were calculated [29,30,31].

Field-emission scanning electron microscopy (SEM, Apreo C, Thermo Scientific, Waltham, USA) coupled with energy-dispersive spectroscopy (EDS, X-Max^N^, Oxford Instruments, Oxford, UK) was used to observe the morphology and analyze the elemental content of residual char after the CONE test, at an accelerating voltage of 20 kV, with a gold-metallized surface. An X-ray diffractometer (X’Pert PRO, PANalytical, Almelo, the Netherlands) with Cu Kα (1.5406 Å) was used to investigate the structure of the residual char. X-ray photoelectron spectroscopy (ESCALAB 250Xi, Thermo Scientific, Waltham, MA, USA) was performed to investigate the elemental status of the residual char.

## 3. Results and Discussion

### 3.1. Microstructure of the Composites

**Figure 1:**
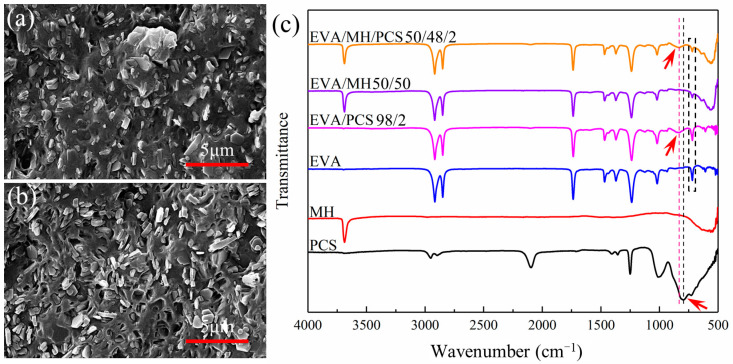
The SEM images of (**a**) EVA/MH 50/50 and (**b**) EVA/MH/PCS 50/48/2; (**c**) the FTIR spectra of the composites.

The microstructure of EVA and its composites are given in Figure 1. Comparing the SEM images of EVA/MH and EVA/MH/PCS, the different dispersion of MH was revealed. For EVA/MH, some aggregations of MH particles can be observed in Figure 1a, even though most of the particles were dispersed uniformly in the matrix. The dispersion of MH particles was remarkably improved for EVA/MH/PCS. As seen in Figure 1b, MH particles were dispersed separately without any aggregations. The results show that PCS enhanced the dispersion of MH particles in the matrix.

In order to explain this effect of PCS, FTIR spectra were analyzed for EVA, PCS, MH, and their composites. According to the spectra in Figure 1c, the absorption peak near 790 cm^−1^ of the Si–C stretching vibration of PCS shifted to 830 cm^−1^ in EVA/PCS or EVA/MH/PCS composites [32,33,34], indicating the interaction between PCS and EVA. Such interaction was probably induced by the formation of hydrogen bonds between Si–H groups in PCS and ester groups in EVA, restricting the stretching of Si–C in the molecular backbone and resulting in the peak shift. Meanwhile, the interaction between PCS and MH was also likely to induce the peak shift. It should be noted that the peak intensity of EVA/MH or EVA/MH/PCS at 720 cm^−1^, corresponding to –CH_2_– bending in the vinyl chain segments of EVA, decreased in comparison to EVA [35], implying the interaction between MH and EVA. The hydrogen bonds between the ester groups in EVA and the hydroxyl groups in MH restricted the bending of methylene adjacent to VA groups. In view of the FTIR results, PCS was found to act as a bridge between EVA and MH, promoting the dispersion of MH particles in the EVA matrix.

### 3.2. Flammability

**Figure 2:**
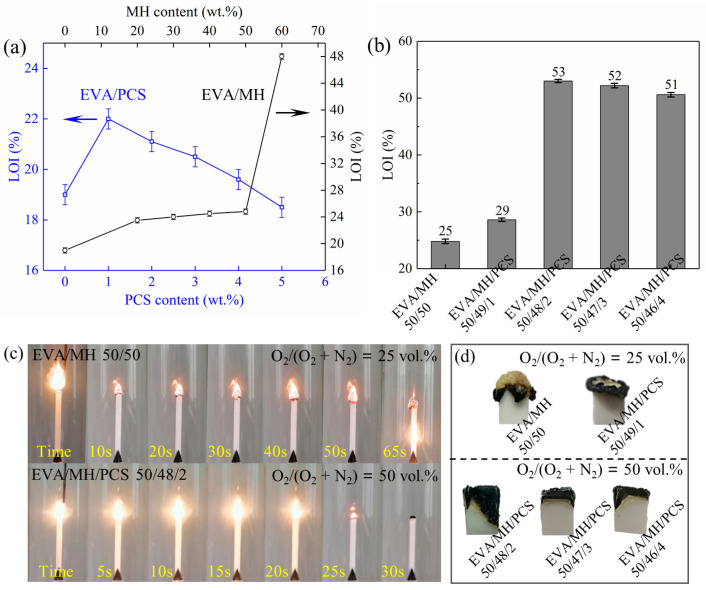
The LOI test results: (**a**) the LOI values of EVA with different PCS and MH content; (**b**) the LOI values of the EVA/MH with different PCS content; (**c**) the burning state during testing of EVA/MH 50/50 and EVA/MH/PCS 50/49/1, and (**d**) the pictures of the extinguished residues after testing.

The flammability based on the LOI was measured for the samples with increasing content of PCS or MH as well as the same overall content of flame retardants (50 wt.%). The LOI values and the burning state as well as the pictures of char of the EVA/MH/PCS composites are shown in Figure 2. The LOI value of EVA was 19, the LOI value of EVA/PCS 99/1 was 22, and the LOI values of EVA/PCS composites decreased with increasing PCS content, as shown in Figure 2a; the LOI value of EVA/PCS 95/5 decreased to 18. Thus, PCS has a negative effect on the flammability of EVA when over a certain content—most likely due to its flammable nature as a polymer. When the MH content was below 50 wt.%, the LOI values of EVA/MH composites increased gradually with increasing MH content. Interestingly, the LOI value of EVA/MH 40/60 sharply increased to 48 from the 25 of EVA/MH 50/50, which was induced by a better structured char building on the top and center of the burning area at high MH content.

The EVA/MH/PCS composites showed higher LOI values than EVA/MH at the same overall flame retardant content of 50 wt.% (Figure 2b). When the content of PCS was as low as 1 wt.%, the LOI of the EVA/MH/PCS 50/49/1 composite was 29. As the content of PCS increased, a remarkable increase in LOI was observed at the PCS content of 2 wt.%. The maximal LOI value of 53 was achieved for the EVA/MH/PCS 50/48/2 composite, representing a 114% increase in comparison with the EVA/MH 50/50 composite. When further increasing the PCS content, the LOI of EVA/MH/PCS decreased slightly, as with EVA/PCS. The EVA/MH/PCS 50/46/4 composite still presented an LOI value as high as 51. Accordingly, the synergistic effect between PCS and MH was revealed, significantly enhancing the flame retardancy of EVA/MN/PCS composites.

During the test, the burning process was recorded by pictures, as shown in Figure 2c, and significant differences were presented during the burning of EVA/MH and EVA/MH/PCS. For EVA/MH, the steady burning stage was observed after ignition, and the char formed around the burning center of the composite, while a new burning interface emerged in the center of the burning area and made it difficult to extinguish. For EVA/MH/PCS, the burning seemed to be fiercer in comparison with EVA/MH, which could be related to the flammable groups in the molecular structure of PCS [36]. A stable char formed tightly on the top of the EVA/MH/PCS composite, and the flame was extinguished simultaneously. The synergistic effect was thus closely associated with the char formed during burning, as shown in Figure 2d. The integrated and compact char played an important role in improving the flame-retardant properties of EVA/MH/PCS.

### 3.3. Combustion Behaviours

**Figure 3:**
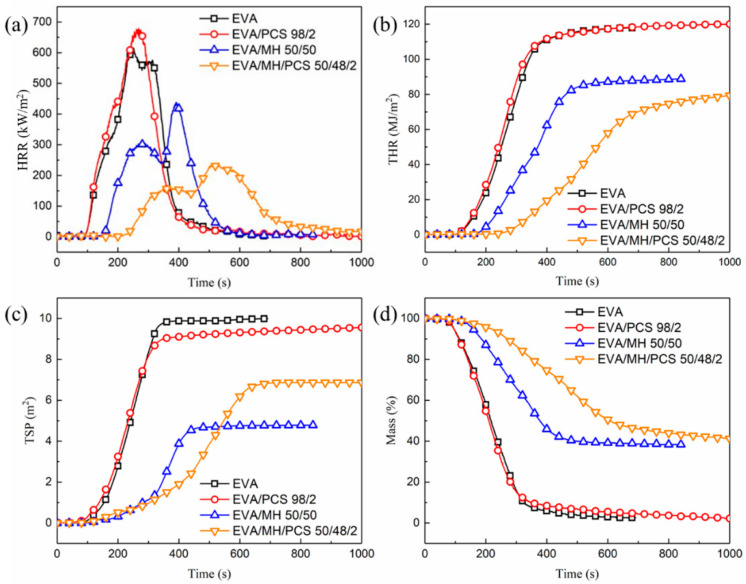
The combustion behaviors of EVA and its composites: (**a**) HRR, (**b**) THR, (**c**) TSP, and (**d**) mass.

The combustion behaviors of EVA and its composites were evaluated using a CONE calorimeter; the results and combustion parameters are shown in Figure 3 and Table 1, respectively. There were no significant differences in the combustion behaviors of EVA and EVA/PCS composites. The TTI and THR of EVA and EVA/PCS were similar to one another. The pHRR and the residual mass of EVA/PCS were a little higher than those of EVA, which was induced by the high heat release and high degradation products left by PCS in condensed phase [26,27]. Moreover, the TSP of EVA/PCS was a little lower than that of EVA. Thus, PCS exerts a limited impact on the combustion behaviors of EVA/PCS 98/2.

When MH was introduced to the composite, the TTI of the EVA/MH/PCS composite increased by 46% (from 149 s for EVA/MH 50/50 to 218 s for EVA/MH/PCS 50/48/2). The HRR (Figure 3a) was effectively inhibited and the THR (Figure 3b) was reduced; there were two heat release peaks for both EVA/MH and EVA/MH/PCS composites, which was related to the formation of the barrier char. Compared with EVA/MH 50/50, the pHRR1 and pHRR2 of EVA/MH/PCS 50/48/2 were decreased by 48% and 46% (Table 1), respectively; moreover, the tpHRR1 and tpHRR2 of EVA/MH/PCS 50/48/2 were prolonged by 18 s and 50 s, respectively. The maximum average heat release rate (MARHE) of EVA/MH/PCS 50/48/2 decreased by 42% compared with EVA/MH 50/50. The smoke generated by EVA resin was inhibited due to the smoke suppression factor of MH [37] (Figure 3c); however, the TSP of EVA/MH/PCS 50/48/2 was a little higher than that of EVA/MH 50/50, due to PCS. The final residual mass was similar, but the mass loss of EVA/MH/PCS 50/48/2 during the combustion procedure was effectively inhibited (Figure 3d). A remarkable synergistic effect between PCS and MH in flame-retardant EVA was revealed by improved combustion behaviors.

According to the combustion parameters, several relevant indices—FPI, FGI, THRI, and TSPI—were also calculated (Table 1) to further evaluate the fire safety performance of the composites. There was no obvious change in EVA/PCS 98/2, indicating that the fire performance of EVA was not affected by PCS. When MH was added, the FPI increased, while the FGI, THRI, and TSPI decreased; thus, the fire performance of EVA was improved by MH. In comparison with EVA/MH 50/50, the FPI of EVA/MH/PCS 50/48/2 increased noticeably (from 0.34 for EVA/MH 50/50 to 0.92 for EVA/MH/PCS 50/48/2), meaning that the EVA/MH/PCS composite was hard to flashover under heat source irradiation. Moreover, significant reductions in TGI and TSPI were apparent—the FGI and TSPI of EVA/MH/PCS 50/48/2 decreased to 0.46 and 0.03 from 1.12 and 0.32 for EVA/MH 50/50, respectively, meaning a greater possibility for people in a fire scenario to escape or be rescued. In short, the fire safety performance of EVA/MH/PCS 50/48/2 improved due to the synergistic effect between PCS and MH.

**Figure 4:**
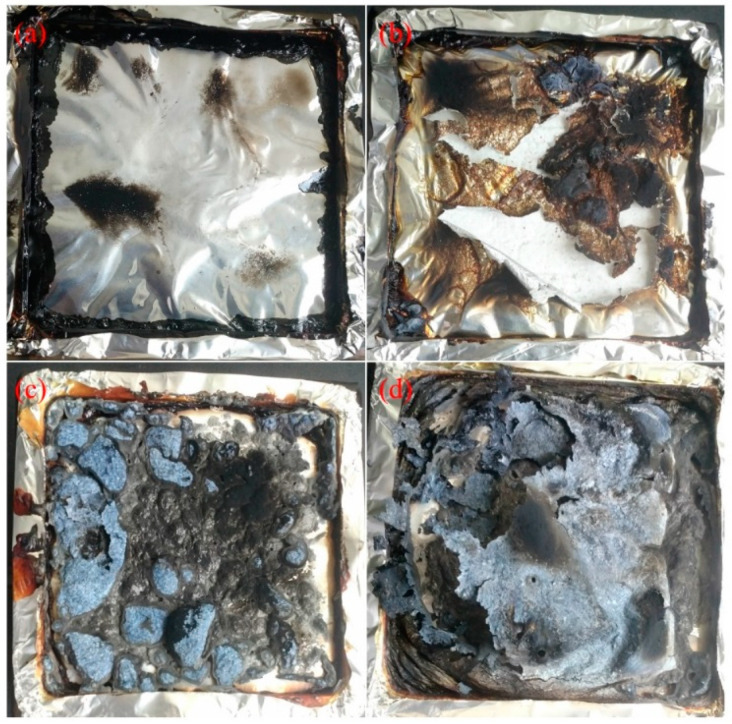
The digital photographs of the residue after the CONE test: (**a**) EVA, (**b**) EVA/PCS, (**c**) EVA/MH, and (**d**) EVA/MH/PCS.

The residual char after the CONE test is shown in Figure 4. There was some tarry residue left after the combustion of EVA (Figure 4a); such tarry residue was easy to form during combustion at relatively low heat flux [5]. Unlike the residue of EVA, the aluminum foil was covered and (Figure 4b) torn apart by the residue of EVA/PCS 98/2, which was related to the shrinkage of the residue induced by ceramization of PCS [38] (the ceramization of PCS is a transformation process from organic linear molecules to an amorphous intermediate phase with a three-dimensional net structure, which is then pyrolyzed to inorganic silicon carbide at higher temperatures; the ceramization of PCS mentioned in this study was revealed in a previous work [27]); this revealed an excellent binding effect of PCS, and the potential to form a cohesive char. The morphology of the residue of EVA/MH/PCS 50/48/2 was different from that of EVA/MH 50/50; a large thin and cohesive char layer covered the surface of the residual char of EVA/MH/PCS 50/48/2 (Figure 4d), which was most likely induced by the binding effect of PCS, while there were some small bubbles in the residue of EVA/MH 50/50 (Figure 4c), indicating the weak binding effect of magnesium oxide.

### 3.4. TG–TFIR Analysis

**Figure 5:**
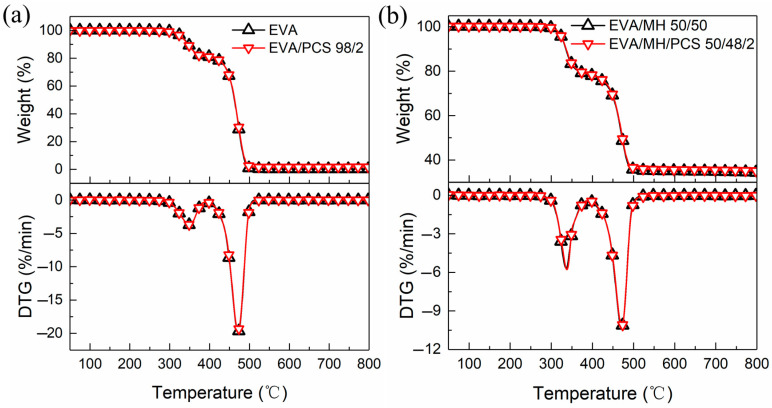
The TG and DTG curves of EVA and its composites: (**a**) EVA and EVA/PCS; (**b**) EVA/MH and EVA/MH/PCS.

Figure 5 shows the TG and DTG curves of EVA and its composites. As can be expected, there are two well-known decomposition steps of EVA: the first step (from 300 °C to 400 °C) is due to the pyrolysis of vinyl acetate groups, while the second step (from 400 °C to 500 °C) is due to the cracking of the polyunsaturated chain generated in the first step [39].

As shown in Figure 5, the TG or DTG curves of EVA/PCS 98/2 and EVA/MH/PCS 50/48/2 were similar to those of EVA and EVA/MH 50/50, respectively, proving that PCS or PCS together with MH exerted no influence on the thermostability of EVA. No significant changes in the degradation parameters of EVA composites with or without PCS were found (Table 2). Only the residue of EVA/PCS increased to some extent in comparison with EVA.

**Figure 6:**
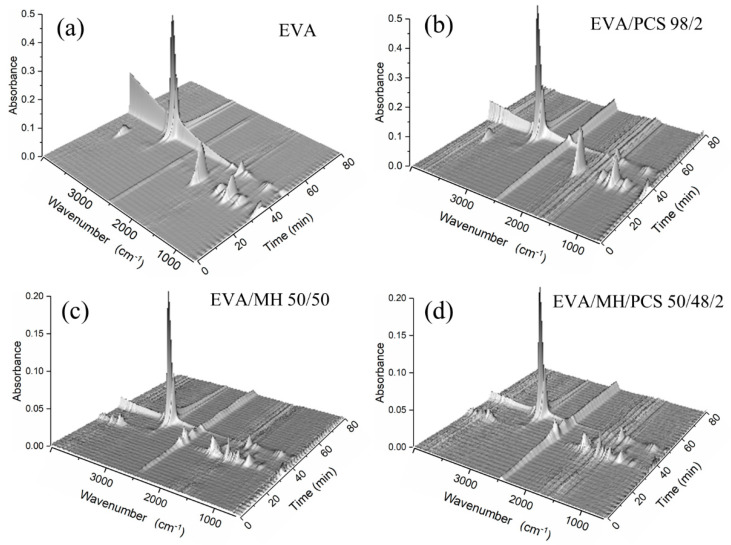
Three-dimensional diagrams of gases evolved in the thermal degradation of EVA and its composites: (**a**) EVA, (**b**) EVA/PCS, (**c**) EVA/MH, and (**d**) EVA/MH/PCS.

**Figure 7:**
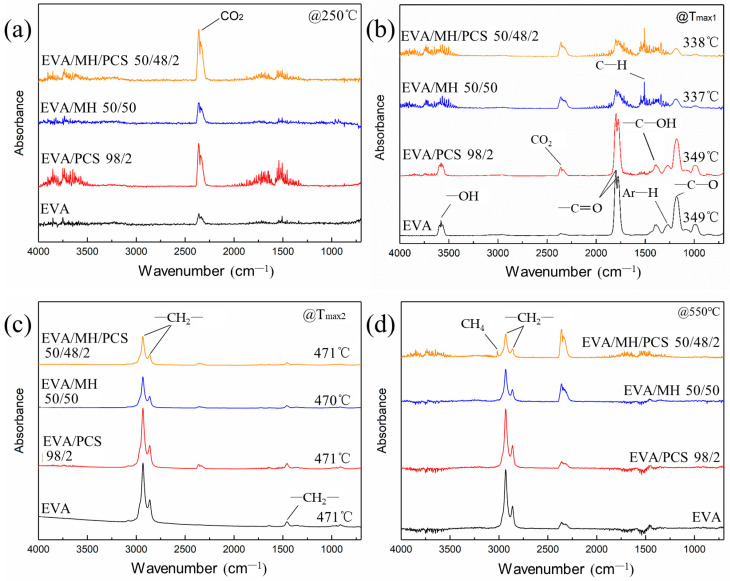
FTIR spectra of the degradation products of EVA and its composites at (**a**) 250 °C, (**b**) Tmax_1_, (**c**) Tmax_2_, and (**d**) 550 °C.

The degradation products in the gas phase of the composites were detected by FTIR; different functional groups of the gas products can be identified from the three-dimensional diagrams, as shown in Figure 6. Two absorbance regions over time were observed for every composite, corresponding to the two degradation steps of the EVA composite. For further details of the gas products, the FTIR curves at the preferred temperature are given in Figure 7, and the details of the absorbance peaks are listed in Table 3.

The absorbance at 2360 cm^−1^ was assigned to carbon dioxide, which was induced by the background atmosphere and was hard to eliminate; this band was obvious at the initial (Figure 7a) and final stages (Figure 7d), where the degradation of EVA composite had not occurred or had finished. The band in the region 960–1000 cm^−1^ arose from plane bending of C–H in alkenes; the bands at 3580 cm^−1^, 1798 cm^−1^, 1385 cm^−1^, and 1175 cm^−1^ belonged to –OH, –C=O, and –C–OH groups, respectively (Figure 7b), due to the degradation products of vinyl acetate groups in the first step [39], while the band at 1509 cm^−1^ was most likely due to asymmetric bending of C–H bonds in methyl [40].

No obvious changes were observed in EVA or EVA/MH 50/50 composites with or without PCS. In terms of the EVA/MH and EVA/MH/PCS 50/48/2 composites, the bands at 3580 cm^−1^, 1798 cm^−1^, 1385 cm^−1^, and 1175 cm^−1^ corresponding to carbonyl or carboxyl became weaker, while the band at 1509 cm^−1^ corresponding to methyl was dominant (Figure 7b), implying that products containing carbonyl or carboxyl were effectively inhibited by MH. Only the bands at 2930 cm^−1^, 2860 cm^−1^, and 1460 cm^−1^ were observed in the FTIR curves of gas released in the second degradation step of EVA composites (Figure 7c), implying that hydrocarbons were the dominant products in this step. There were still infrared absorbance peaks of methyl and methylene in the final stage (Figure 7d), corresponding to the further degradation of the residue char. As expected, the band at 3019 cm^−1^ (C–H stretching in methane) occurred in the final degradation products of EVA/MH/PCS 50/48/2, while this was not of the case with EVA/PCS 98/2—probably due to the interaction between the tarry residue of EVA and the residue of PCS, which inhibited the production of methane; this interaction became weak with the presence of active magnesium oxide, which had catalytic effect on the carbonaceous residue [37].

### 3.5. Identification of Pyrolyzates by Py–GC/MS

**Figure 8:**
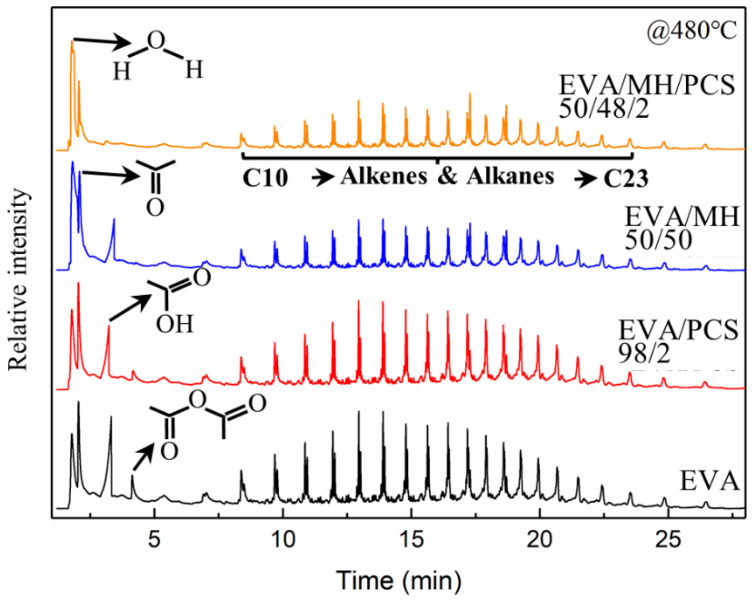
The pyrograms of EVA and its composites obtained from Py–GC/MS analysis at 480 °C.

The EVA and its composites were pyrolyzed at 480 °C, where both vinyl acetate units and polyethylene units would decompose simultaneously; the resulting pyrograms are summarized in Figure 8. There were similar alkene and alkane products generated from EVA and its composites, and four kinds of small molecule substances were found in the pyrolyzates of EVA and EVA/PCS composites—namely, water, acetone, acetic acid, and acetic anhydride, which were derived from vinyl acetate units [41]. Meanwhile no acetic anhydride was found in the pyrolyzates of EVA/MH 50/50, and no acetic anhydride or acetic acid were found for EVA/MH/PCS composites.

There was acetone generated in all EVA composites; thus, the ratio of other gas products to acetone was adopted in order to evaluate the relative content of the products. The ratios of relative intensity of acetic anhydrate to acetone and acetic acid to acetone were 33% and 86%, respectively, for EVA, while they were 20% and 61%, respectively, for EVA/PCS 98/2, and the ratio of acetic acid to acetone for EVA/MH 50/50 was 53%, implying that the formation of acetic acid was inhibited in the presence of PCS or MH, as shown in Figure 7b; the acetic anhydride was eliminated in the presence of MH, while this was not the case for PCS. This phenomenon was probably due to the interaction generated between active magnesium oxide and acetic acid [42], which was further intensified by PCS, as no acetic acid could be found in pyrolyzates derived from EVA/MH/PCS 50/48/2; thus, a remarkable synergistic effect between MH and PCS on the thermal pyrolysis of EVA composites was proven here.

### 3.6. Morphology of the Char

**Figure 9:**
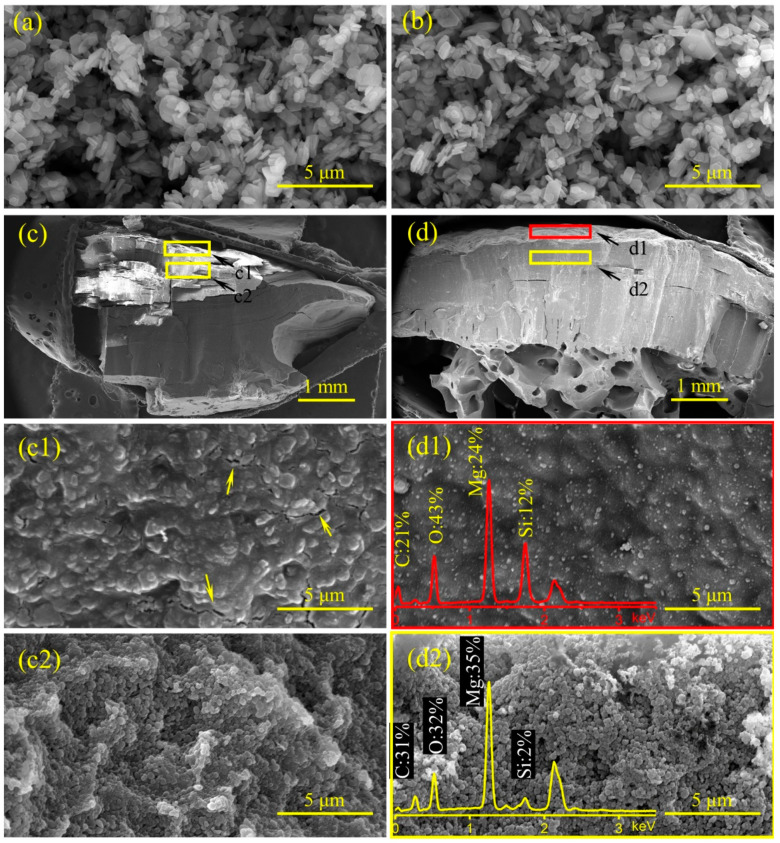
SEM micrographs of MH (**a**), MgO (**b**), the residual char of EVA/MH 50/50 (**c**,**c1**,**c2**), and EVA/MH/PCS 50/48/2 (**d**,**d1**,**d2**) after the CONE test: the cross-section at 60× magnification (**c**,**d**), the surface (**c1**,**d1**), and the cross-section at 20,000× magnification (**c2**,**d2**); the EDS spectra were embedded in the corresponding SEM micrographs.

Based on the HRR curves of EVA/MH 50/50 and EVA/MH/PCS 50/48/2, the EVA/MH/PCS 50/48/2 composite exhibited a better barrier effect. Here, the influence of PCS on the char structure was investigated in order to disclose the mechanism of the improved barrier effect. The residual char after the CONE test and MH as well as magnesium oxide (MH heat treated at 500 °C for 30 min in air) were characterized by SEM coupled with EDS, as shown in Figure 9.

The morphologies of MH (Figure 9a) and MgO (Figure 9b) were approximate hexagonal plates, indicating that the hexagonal platelet structure of MH particles was not changed during dehydration under heat treatment [43]. The char structure with macroscale size in the cross-section of EVA/MH 50/50 (Figure 9c) was compact, but there were many parallel cracks in the char body, and it was easy to divide into small pieces along the parallel direction. In comparison with EVA/MH 50/50, the char structure in the cross-section of EVA/MH/PCS 50/48/2 (Figure 9d) was cohesive and compact, and a special porous structure was formed in the bottom of the char along the thickness direction. There were many cracks in the surface char of EVA/MH 50/50 (marked by an arrow in Figure 9c1), while no cracks were found in the surface char of EVA/MH/PCS 50/48/2 (Figure 9d1), implying that the barrier effect of the char was improved by PCS.

The silicon atomic percentage along the thickness direction was analyzed by EDS. The corresponding positions in the char for EDS analysis are marked by the red rectangle (surface area) and yellow rectangle (body area) in Figure 9d; the actual area for EDS analysis is presented in Figure 9d1 (surface area by red rectangle) and Figure 9d2 (body area by yellow rectangle). The silicon atomic percentage in the surface (Figure 9d1) and inner section (Figure 9d2) of EVA/MH/PCS 50/48/2 was ~12% and 2%, respectively; an accumulation phenomenon of silicon in the surface char was revealed, proving that the migration of PCS to the surface took place during the combustion. The migration of PCS from the inner section to the surface led to more PCS accumulation in the burning interface, which was beneficial to forming a compact and cohesive char layer, as more PCS participated in the organic–inorganic transition.

It should be noted that the morphology of MgO particles in the residual char of EVA/MH 50/50 (Figure 9c2) and EVA/MH/PCS 50/48/2 (Figure 9d2) was different from that of MH (Figure 9a) or MgO (Figure 9b). MgO in the char showed a roughly spherical shape instead of its original hexagon plates; referring to our previous study of PE/MH/PCS [27], this phenomenon is likely related to the degradation of vinyl acetate groups in EVA, which induced the dehydration of MH at lower temperatures.

### 3.7. Mechanistic Analysis

**Figure 10:**
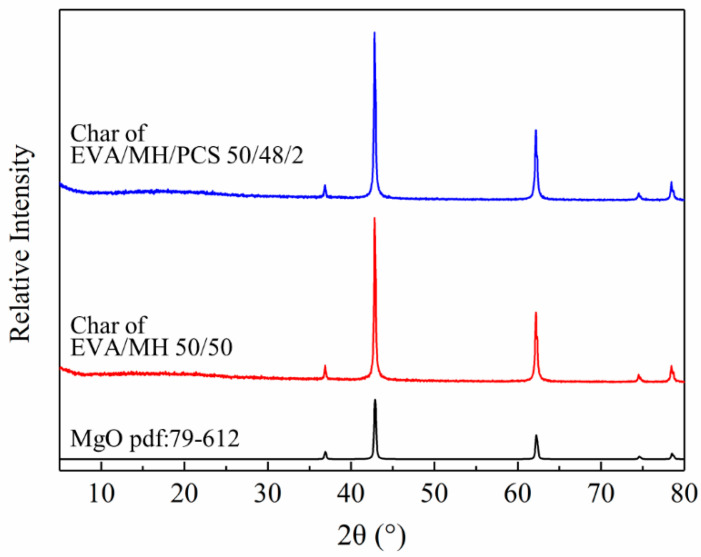
XRD patterns of MgO and the residual char of EVA/MH and EVA/MH/PCS.

**Figure 11:**
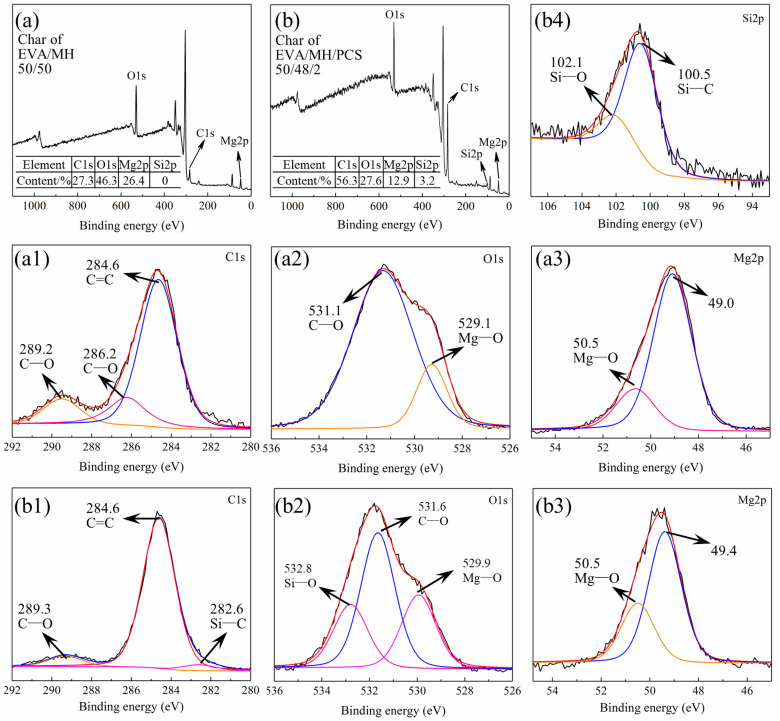
The XPS spectra of the surface char of (**a**) survey spectrum of EVA/MH and (**b**) survey spectrum of EVA/MH/PCS. (**a1**,**b1**) C1s, (**a2**,**b2**) O1s, (**a3**,**b3**) Mg2p, and (**b4**) Si2p.

The surface char was collected from the residue char after the CONE test and investigated by XRD. As seen in Figure 10, all samples showed the same diffraction peaks with magnesium oxide (pdf card: 79-612), confirming that magnesium oxide was the main product after the combustion of the composites. The bonding state of the elements in the residue char of the EVA/MH 50/50 and EVA/MH/PCS 50/48/2 composites was analyzed by XPS, as shown in Figure 11.

The residual char of EVA/MH 50/50 contained C, O, and Mg, the contents of which were 27.3%, 46.3%, and 26.4%, respectively, as shown in Figure 11a. The bonding states of C1s, O1s, and Mg2p are given in Figure 11a1–a3. The bonding state of C1s at 289.2 eV (Figure 11a1) or O1s at 531.1 eV (Figure 11a2) was C–O in carbonates, implying the formation of magnesium carbonate in the surface of the MgO particles [44,45]. The bonding state of C1s at 286.2 eV (Figure 11a1) was C–O in aromatic carbon [46], confirming the formation and the oxidation of carbon coke generated from EVA.

Additionally, Si was detected in the residual char of EVA/MH/PCS 50/48/2, as shown in Figure 11b; the contents of C, O, Mg, and Si were 56.3%, 27.6%, 12.9%, and 3.2%, respectively. Significantly, the content of C in the residual char of EVA/MH/PCS 50/48/2 was two times higher in comparison with EVA/MH 50/50; more carbon accumulated in the surface of the residue due to PCS, in accordance with the EDS results shown in Figure 9. The bonding states of C1s, O1s, Mg2p, and Si2p were analyzed, and are shown in Figure 11b1–b4, respectively. It should be noted that the bonding state of C1s at 286.2 eV was not found in the residue of EVA/MH/PCS 50/48/2 (Figure 11b1); that is, the oxidation of carbon coke was inhibited by PCS—probably due to the formation of Si–O and Si–C products. The bonding states of C1s at 282.6 eV (Figure 11b1) and Si2p at 100.5 eV (Figure 11b4) belonged to Si–C in amorphous products generated by PCS [47]. The bonding states of O1s at 532.8 eV (Figure 11b2) and Si2p at 102.1 eV (Figure 11b4) belonged to Si–O in silicates [48]. Thus, the magnesium silicate formed in the surface of MgO particles in the residue (as proven in our previous work [27]), which could act as a bridge for binding MgO particles together, and this was more effective than magnesium carbonate formed in the surface of MgO particles. This could account for the sharp increase in the LOI values of EVA/MH 40/60 shown in Figure 2a, as well as the effective improvement of the LOI values of EVA/MH/PCS composites shown in Figure 2b. In short, PCS exerted a noteworthy physical barrier effect of the residue on flame-retardant EVA/MH composites.

**Figure 12:**
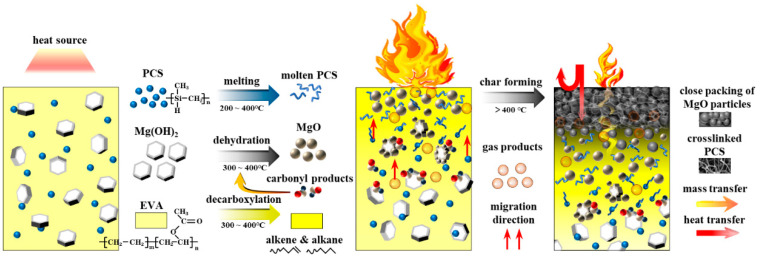
The schematic diagram of the char formation process of the EVA/MH/PCS composite.

The char formation process is elaborated in Figure 12. The decomposition reaction of MH proceeded from the edge of the plate or cracks in the interface towards the center of the crystal [49]. The acetic acid generated by decarboxylation of vinyl acetate groups in EVA matrix was more likely to interact with MH and lead to the formation of spherical MgO particles [50]. Spherical MgO particles tend to form close packing. The intermediate products of PCS interacted with MgO and magnesium silicate formed in the surface of MgO particles, which acted as a binder between the intermediate products of PCS and MgO particles; the intermediate products of PCS also tended to form a three-dimensional net structure, which could gather MgO particles effectively. Thus, a compact and cohesive char formed.

## 4. Conclusions

The combustion behaviors and fire performance of EVA/MH/PCS composites were investigated, and the results indicate that PCS has a dramatic synergistic effect with MH in flame-retardant EVA. The LOI and the combustion behaviors of EVA/MH/PCS were significantly improved due to PCS; the thermostability of EVA/MH/PCS composites was not improved, but the degradation behavior was affected by PCS, as acetic acid and acetic anhydride were inhibited in EVA/PCS degradation products or in the pyrolysis products of EVA/MH/PCS. The hexagonal shape of MH particles turned into the spherical shape of MgO particles during dehydration, due to the VA groups in EVA. The improved physical barrier effect of the residue was the dominant mechanism in improving the combustion behaviors and flame-retardant properties of the EVA/MH/PCS composites, which was achieved by the organic–inorganic transition of PCS and the interfacial reaction between MgO and PCS, forming magnesium silicate.

## Figures and Tables

**Table 1:** The combustion parameters of EVA and its composites.

Sample	EVA	EVA/PCS	EVA/MH	EVA/MH/PCS
TTI (s)	91	92	149	218
tpHRR1 (s)	153	176	128	146
pHRR1 (kW/m^2^)	619	678	307	160
tpHRR2 (s)	--	--	243	293
pHRR2 (kW/m^2^)	--	--	438	236
THR (MJ/m²)	118	120	88	85
TSP (m^2^)	10	9.5	4.7	6.8
FPI (s·m^2^/kW)	0.15	0.14	0.34	0.92
FGI (kW/(m^2^·s))	2.54	2.53	1.12	0.46
THRI (MJ/m²)	2.05	2.05	1.92	1.67
TSPI (m^2^)	0.75	0.69	0.32	0.03

**Table 2:** The TG and DTG parameters of EVA and its composites.

Sample	T_5_ (°C)	T_10_ (°C)	T_50_ (°C)	T_max1_ (°C)	T_max2_ (°C)	R_max1_ (%/min)	R_max2_ (%/min)	Residue at 600 °C (%)
EVA	332	347	462	349	471	−3.6	−19.7	0.3
EVA/PCS 98/2	331	342	463	349	471	−3.6	−19.4	1.5
EVA/MH 50/50	326	335	472	337	470	−5.6	−10.3	35.1
EVA/MH/PCS 50/48/2	326	336	473	338	471	−5.7	−10.2	35.6

**Table 3:** Assignments of the IR bands to vibrational modes of atomic groups.

Group	Vibration Mode	Wavenumber (cm^−1^)
–OH	O–H stretching	3580
CO_2_	C=O stretching	2360
CH_4_	C–H stretching	3015
–CH_2_–, –CH_3_	C–H asymmetric stretching	2930
–CH_2_-, –CH_3_	C–H symmetric stretching	2860
–C=O	stretching	1798
–CH_3_	C–H asymmetric bending	1509
–CH_2_-	scissoring	1460
–C–OH	C–O in-plane bending	1385
Ar–H	C–O stretching	1265
–C–OH	C–O stretching	1175
=CH_2_	=C–H out of plane bending	960–1000
